# Cancer-associated fibroblasts strengthen cell proliferation and EGFR TKIs resistance through aryl hydrocarbon receptor dependent signals in non-small cell lung cancer

**DOI:** 10.1186/s12885-022-09877-7

**Published:** 2022-07-13

**Authors:** Hao Feng, Boxiong Cao, Xuan Peng, Qiang Wei

**Affiliations:** The First People’s Hospital of Shuangliu District, Chengdu (West China Airport Hospital of Sichuan University), Near 51 Kangle Lane, Chengdu City, 610200 Sichuan Province China

**Keywords:** Cancer-associated fibroblasts, EGFR TKIs resistance, Kynurenine, Aryl hydrocarbon receptor, Non-small cell lung cancer

## Abstract

The tumor microenvironment is a dynamic cellular milieu that interacts with cancer cells and promotes tumor progression and metastasis. However, the specific mechanisms by which the tumor microenvironment impacts cancer cells’ behaviors remain poorly understood. In this study, enriched cancer-associated fibroblasts (CAFs) were observed in tumor tissues isolated from epidermal growth factor receptor tyrosine kinase inhibitors (EGFR TKIs) resistant non-small cell lung cancer (NSCLC) patients. CAFs isolated from tumor tissues were capable of producing tryptophan metabolite kynurenine (Kyn), which significantly increased the proliferation and EGFR TKIs resistance of NSCLC cells. In this study, it was further observed that the activation of tryptophan 2,3-dioxygenase (TDO) in CAFs, resulted in the enhanced capability of tryptophan metabolism in them compared to normal fibroblasts. As a result, Kyn produced by CAFs facilitated the up-regulation of Aryl Hydrocarbon Receptor (AhR) signals in NSCLC, thereby resulting in the downstream ATK and ERK signaling pathways activation. Finally, inhibition of AhR signals efficiently prevented tumor growth and development of EGFR TKIs resistance, eventually improved the outcome of EGFR TKIs, and described a promising therapeutic strategy for NSCLC.

**Supplementary Information:**

The online version contains supplementary material available at 10.1186/s12885-022-09877-7.

## Introduction

Being the most common type, NSCLC accounts for about 75% of lung cancers, the leading cause of carcinoma-associated death worldwide, and patients with NSCLC exhibit a low 5 years survival rate of less than 15% [1]. The vast majority of patients who suffered from the drugs resistance or tumor recurrence following clinical interventions, usually progressed to high degree stages of carcinoma, along with distant metastasis and invasion into surrounding tissues [2]. Patients with NSCLC have a high potential to develop chemotherapy resistance that could be linked to complex signaling networks of tumor cells and their crosstalk with the microenvironments [3]. Hence, there is an urgent demand to elucidate the underlying mechanisms of NSCLC progression eventually that will help develop innovative strategies to impair tumor progression.

Recently, the clinical application of EGFR TKIs [4], anaplastic lymphoma kinase inhibitors [4], and ROS-associated kinase inhibitors [5, 6] remarkably extended the progression-free survival of NSCLC patients. However, in most cases, because of the acquired drug resistance of EGFR to EGFR TKIs, the EGFR targeted therapeutic strategy failed to prolong the survival time of NSCLC patients [7]. The development of drugs resistance is bound up with diverse biological processes, such as the up-regulation of ATP-binding cassette transporters, the occurrence of tumor dormancy, activation of anti-apoptosis proteins, and the crosstalk between tumor cells and their microenvironment [8]. Recent pieces of evidence suggest that elements derived from the tumor microenvironment participate in the tumor progression and are closely linked to the development of multi-drug resistance in several tumor types [9, 10]. Among the compounds of the tumor microenvironment, CAFs are emerging as critical factors that are involved in tumor progression and promotion of multi-drug resistance [11]. CAFs are spindle-shaped mesenchymal cells that reside inside the tumor tissue and have phenotypes of fibroblasts and smooth muscle cells. Recent studies have demonstrated that through the secretion of cytokines and matrix metalloproteinases, CAFs regulate diverse tumor behaviors, such as tumor growth [12] and distant metastasis [13]. Furthermore, CAFs are reported to be involved in the development of tumor drugs resistance in breast and colorectal cancers [14]. However, the underlying mechanism of EGFR TKIs resistance in lung cancer is excursive, and the potential role of CAFs in lung cancer remains controversial.

In this study, enriched CAFs distribution was observed in EGFR TKIs resistant tumor tissues isolated from NSCLC patients. Activation of TDO and elevated secretion of Kyn in CAFs isolated from NSCLC patients revealed that CAFs are closely linked to the lung cancer cells proliferation and EGFR TKIs resistance. This study further provided evidence that CAFs produced Kyn to facilitate EGFR TKIs resistance through regulation of AhR/AKT/ERK signaling pathway. Finally, the combination of AhR inhibitor and EGFR TKIs showed a significant suppressive effect on tumor growth and reversed the EGFR TKIs resistance. All these findings are suggesting a combination of AhR inhibitor and EGFR TKIs as a novel therapeutic strategy to treat lung cancers.

## Materials and methods

### Cell lines culture and regents

Human lung cancer cells A549, murine lung cancer cells Lewis, human embryonic lung fibroblasts HFL1, murine NIH-3 T3 fibroblasts were obtained from Cell Bank of Chinese Academy of Sciences (Shanghai, China). All cell lines were cultured in RPMI-1640 (Gibico, MA, USA) complete culture medium supplemented with 10% fetal bovine serum (Gibco, MA, USA). Erlotinib (Erl) and gefitinib (Gef) were from Sangon (Shanghai, China). AhR inhibitors DMF, AKT inhibitor MK-2206, ERK inhibitor ravoxertinib (Rav) and Kyn were derived from MCM (NJ, USA).

## Lung cancer patients’ tumor tissues collection

NSCLC tumor samples were obtained after the surgery sterilely at Chengdu Shuangliu District First People’s Hospital. All samples were reviewed according to World Health Organization classification by the pathologist. All patients were treated with surgical resection combined with adjuvant TKI inhibitors treatment (1st, 2nd generations). Samples were divided into D-R and D-S groups according to follow up visit (recurrent in 3 years, D-R and non-recurrent in 3 years, D-S). Samples were collected and processed in accordance with the declaration of Helsinki. And this study obtained the ethical approval from the Chengdu Shuangliu District First People’s Hospital.

### CAFs isolation and culture

Tumor tissues isolated from patients or Lewis-bearing C57BL/6 mice were collected and cut into pieces as small as possible. Then tissues were digested by DMEM culture medium (Gibco, Thermo, MA, USA) containing ACCUMAXTM (SIGMA, MA, USA) at 37 °C, 5% CO2 incubator for 2 hours, followed by filtration (40 μm, Thermo, MA, USA). On one hand, the cells suspension was stained by primary antibody to CD45 and CD90 (eBioscience, MA, USA) for CAFs percentage analysis. The CD45+/CD90+ cells were sorted using fluorescence-activated cell sorting for CAFs quantification. On the other hand, the collected cell suspensions were seeded into 6-well plate containing 2 ml RPMI-1640 culture medium with 10% fetal bovine serum overnight at 37 °C. After 12 hours, fresh medium was used to remove the un-adherent cells and collect the remaining cells. After 5 passages, we sorted the CD45+/CD90+ positive CAFs. The CD45+/CD90+ cells were identified as CAFs, which would be identified by α-SMA analysis before cells co-culture. The CD45−/CD90- cells were identified as tumor cells for α-SMA negative control. The isolated CAFs were cultured in 1640 complete culture medium with 10% fetal bovine serum at 37 °C, 5% CO2 incubator for at most 20 passages.

### Cell proliferation analysis

Cell proliferation was detected using the CCK8 kit (Solarbio, Beijing, China). Briefly, 1 × 10^3^ cancer cells were seeded into 96-well culture plates. In determining time points, 20 μl of CCK-8 solution was added to the 96 wells. After incubation for 2 hours, at 37 °C and 5% CO2 the absorbance was measured at 450 nm using a microplate reader (Bio-Rad, MA, USA). Each experiment was performed independently three times [15].

### Cytotoxicity analysis

The cytotoxicity was analyzed using the FITC-Annexin V/ PE-PI apoptosis detection kit (BD, NJ, USA). Briefly, agents treated tumor cells were resuspended and stained with FITC-Annexin V and PE-PI staining solution for 15 min. Early, the presence of late apoptotic and necrotic tumor cells was determined as cell apoptosis. Then cells apoptosis was detected by a C6 flow cytometer (BD, NJ, USA). Each experiment was repeated three times independently [15].

### Western blotting

Protein samples (20 μg) in cells lysis buffer were separated by SDS-PAGE, transferred to PVDF membranes, and incubated with primary antibodies for α-SMA (1:800; Abcam, Cambridge, UK), IDO1 (1:500; Abcam, Cambridge, UK), TDO2 (1:500; Abcam, Cambridge, UK), Kyn (1:800; Abcam, Cambridge, UK), total AKT (1:500; Abcam, Cambridge, UK), p-AKT (1:500; Abcam, Cambridge, UK), total ERK1/2 (1:500; Abcam, Cambridge, UK), p-ERK1/2 (1:500; Abcam, Cambridge, UK), or actin (1:1000; Abcam, Cambridge, UK) at 4 °C overnight [15]. Samples were then incubated with HRP-conjugated secondary antibody (1:800; Abcam, Cambridge, UK) for 1 hour at room temperature [15]. The ECL detection kit (Thermo, MA, USA) was used for the visual detection of targeted proteins. All the images of the Western blot are provided as Supplementary information.

### Immunofluorescent staining

A549 cells were seeded on confocal dishes and fixed with 4% paraformaldehyde for 15 min, then permeabilized with 0.5% Triton X-100 for 10 min. Samples were blocked with blocking solution containing PBS and 5% bovine serum albumin for 30 min, and stained with anti-AhR primary antibodies (1:200; Abcam, Cambridge, UK) at 4 °C overnight, followed by incubation with secondary antibodies (1:1000; Abcam, Cambridge, UK) for 1 hour at room temperature. The samples were captured under an Olympus confocal microscope (Tokyo, Japan) and the fluorescence intensity was analyzed by image J software 1.8.0 (NIH, Bethesda, Maryland, USA).

### Enzyme-linked immunosorbent assay (ELISA)

Kyn quantification in supernatant of HFL1 or CAFs was determined by a human Kyn ELISA kit (FineTest, China). Briefly, 5 × 10^4^ fibroblasts were seeded in 24-well plate containing 1 ml culture medium. After 48 hours, supernatant was collected and Kyn concentration was determined by ELISA kit according to protocol. Each experiment was repeated three times independently.

### Animal protocols

Female C56BL/6 mice and NOD-SCID mice aged 6 to 8 weeks were purchased from Huafukang (Beijing, China), and placed in a specific pathogen-free facility. All animal experiments were performed according to the guidelines approved by the Institute Ethics Committee of Chengdu Shuangliu District First People’s Hospital. For the subcutaneous lung cancer mice model, 10^6^ A549 cells (50 μl PBS) were subcutaneously injected into NOD-SCID mice and 10^5^ Lewis cells (50 μl PBS) were subcutaneously injected into C56BL/6 mice. When tumor volume reached 800 mm^3^, mice were treated with PBS, Gef (20 mg/kg), Erl (25 mg/kg), Gef or Erl combined with DMF (intragastric injection of 10 mg/kg) twice a week. For the Kyn persistence test, A549 bearing mice were treated with an intratumor injection of Kyn (10 μM in 50 μL PBS) once a week to mediate the drug resistance. The tumor volumes of mice were recorded at every 5 days interval. Survival was recorded daily (*n* = 6). The tumor volume was calculated using the following formula:

tumor volume = length × width ^2^/2.

### Statistical analysis

Each experiment was performed for at least three independent times. Results were presented as the mean ± SD and the statistical significance was analyzed using GraphPad 6.0 software (La Jolla, CA, USA). Statistical significance between groups was calculated by Student’s t test for two groups or by one-way ANOVA for more than two groups. The survival rates were determined by Kaplan–Meier survival analysis, and *p* < 0.05 was considered as a significant difference.

## Results

### CAFs enhance lung cancer cells proliferation and EGFR TKIs resistance

**Fig. 1:**
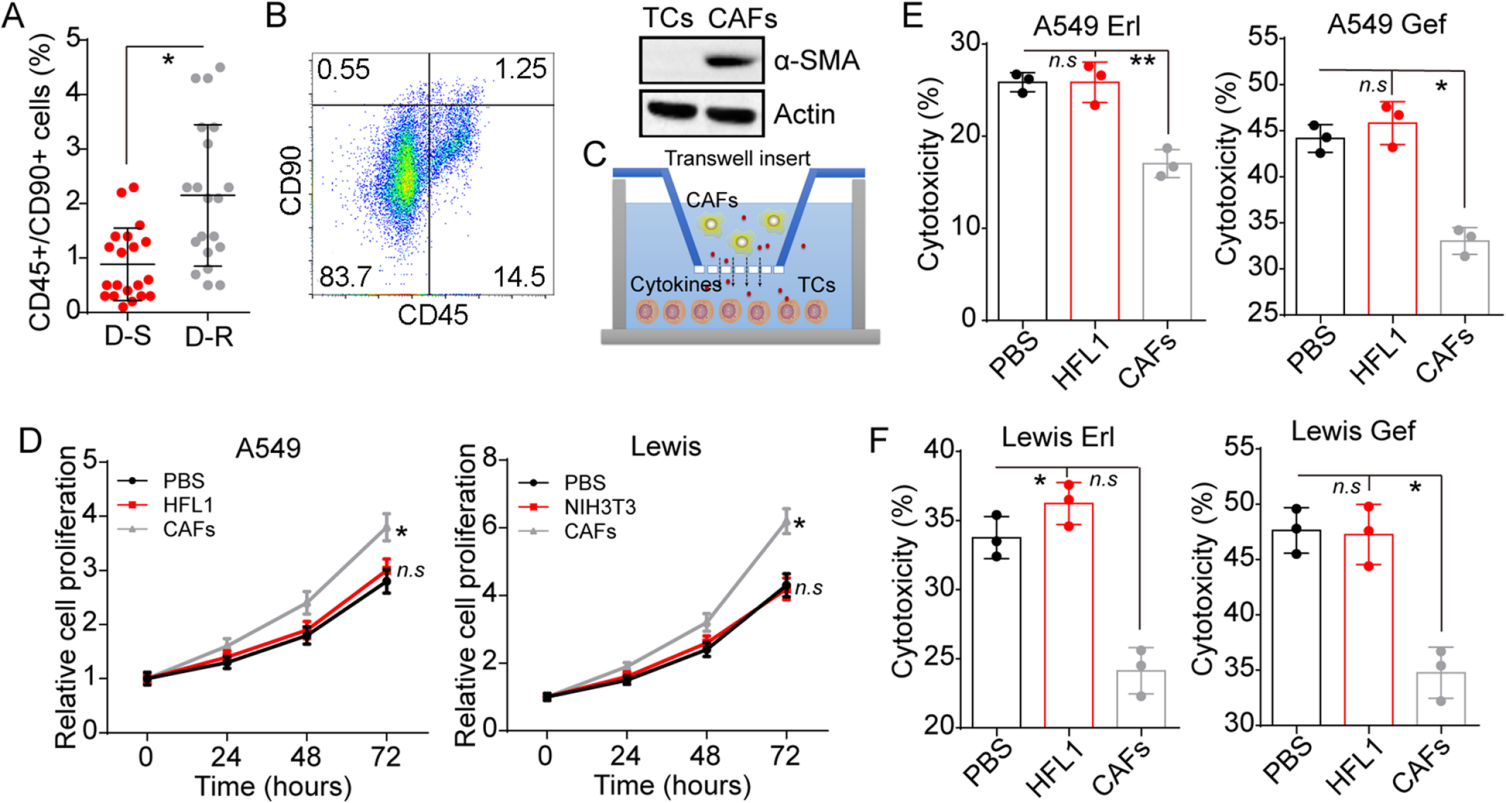
CAFs mediate lung cancer cells proliferation and EGFR TKIs resistance. **A**, the percentage of CD90 positive cells in the CD45+ cells subpopulation isolated from lung cancer patients’ tumor tissues, which is divided into drug-resistant (D-R) and drug-sensitive (D-S) groups (*n* = 20). **B**, CD45+/CD90+ cells sorting using flow cytometry from tumor tissues, and Western blotting of α-SMA in tumor cells (TCs) and CD45+/CD90+ CAFs isolated from NSCLS patients’ tumor tissues. **C**, the schematic diagram of tumor cells and CAFs co-culture system. **D**, the relative cells proliferation of A549 cells pre-cultured with PBS, HFL1, and CAFs isolated from patients (fibroblast: tumor cells, 1:5, 48 hours). the relative cells proliferation of Lewis cells was pre-cultured with PBS, HFL1, and CAFs isolated from C57BL/6 (fibroblast: tumor cells, 1:5, 48 hours). **E**, cytotoxicity of A549 cells pre-cultured with PBS, HFL1, CAFs to Erl (5 μM, 48 hours) and Gef (10 μM, 48 hours). **F**, cytotoxicity of Lewis cells pre-cultured with PBS, NIH-3 T3, CAFs to Erl (5 μM, 48 hours) and Gef (10 μM, 48 hours). * indicates *P* < 0.05. ** indicates *P* < 0.01. n.s. indicates no statistical significance

Current studies have suggested that CAFs in the extracellular matrix served as a critical participant in tumor progression. To further determine the potential role of CAFs in EGFR TKIs resistance development, we analyzed the proportion of CD45+/CD90+ cells subpopulation, which is identified as CAFs, in tumor tissues from EGFR TKIs sensitive/resistant NSCLC patients (Fig. S1A). Intriguingly, an increased percentage of CD45+/CD90+ cells was observed in the drug-resistant (D-R) group compared to the drug-sensitive (D-S) group (Fig. 1A). To further confirm these sorted CAFs, we isolated those CD45+/CD90+ cells by fluorescence-activated cell sorting and examined the expression of α-SMA (a marker of fibroblasts). Intensive α-SMA expression in the CD45+/CD90+ cells compared to CD45−/CD90- (Fig. 1B). These results implicated that EGFR TKIs resistant lung cancer tissues possess a higher proportion of CAFs, which might be associated with the development of EGFR TKIs resistance in lung cancer. Based on this point, we seeded tumor cells into a 24-well plate with a transwell insert (3 μm) containing CAFs (Fig. 1C).

Notably, CAFs co-cultured NSCLC cells (A549 and PC-9) showed increased proliferation potential compared to the control group, whereas the same phenomenon could not be observed in normal human fibroblasts cells HFL1 (Fig. 1D and S1B). Similarly, Lewis tumors derived CAFs also significantly increased murine lung cancer cells proliferation (Fig. 1D), suggesting that CAFs could mediate cell proliferation and tumor sustained growth in lung cancer. Additionally, tumor tissues derived CAFs significantly enhanced A549/PC-9 cells’ resistance to EGFR TKIs namely Erl and Gef (Fig. 1E and S1C). And the same results were observed in murine CAFs treated with Lewis cells (Fig. 1F). These results implicated that CAFs could upregulate lung cancer cells proliferation and EGFR TKIs resistance, resulting in poor therapeutic outcomes.

### CAFs produce Kyn to promote lung cancer cell proliferation and resistance to Gef/Erl

**Fig. 2:**
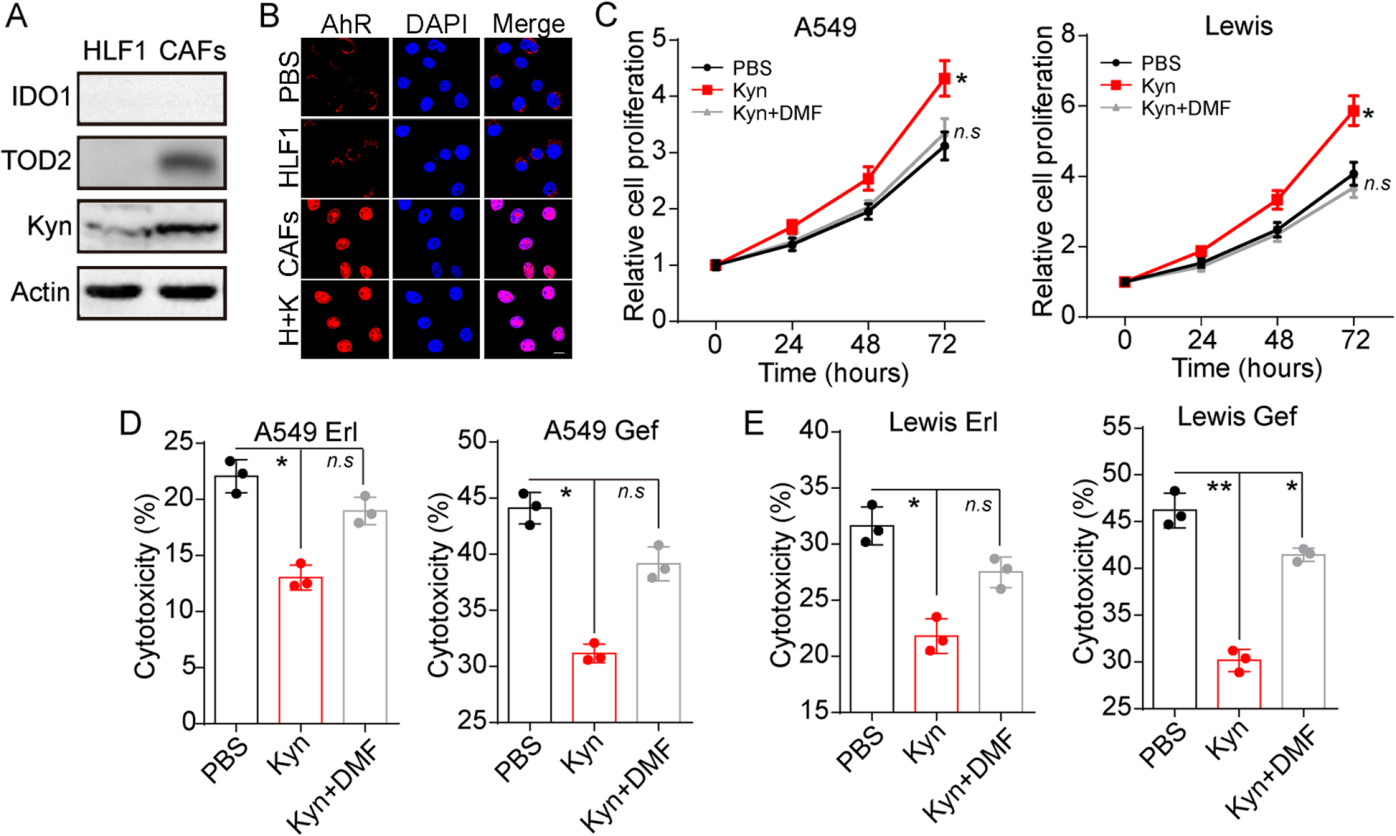
CAFs up-regulated AhR signals in lung cancer cells through Kyn. **A**, Western blotting of IDO1, TDO2, Kyn in HLF1 and CAFs isolated from lung cancer patients. **B**, the immunofluorescence of AhR in A549 cells cultured with PBS, HFL1, CAFs, HFL1 combined with Kyn (fibroblast: tumor cell, 1:5, Kyn 100 μM, 48 hours). The scale bar is 10 μm. **C**, the relative cells proliferation of A549 and Lewis treated with PBS, Kyn (100 μM), and Kyn (100 μM) combined with DMF (10 μM). **D**, the cytotoxicity of A549 pre-treated with PBS, Kyn, and Kyn combined with DMF (Kyn 100 μM, DMF 10 μM, 48 hours) to Erl (5 μM, 48 hours) and Gef (10 μM, 48 hours). **E**, the cytotoxicity of Lewis pre-treated with PBS, Kyn, and Kyn combined with DMF (Kyn 100 μM, DMF 10 μM, 48 hours) to Erl (5 μM, 48 hours) and Gef (10 μM, 48 hours). * indicates *P* < 0.05. ** indicates *P* < 0.01. n.s. indicates no statistical significance

Next, we wondered how CAFs strengthened EGFR TKIs resistance in lung cancer. Compelling findings illustrated that CAFs could facilitate tumor drugs resistance through the secretion of diverse cytokines or metabolites. Among those, Kyn is a metabolite of the amino acid tryptophan through the IDO/TDO metabolic enzymes. And the expression of Kyn has been reported to be strictly correlated to the tumor progression [16]. Intriguingly, up-regulation of TDO expression was found in CAFs compared to the normal fibroblast HLFs, and no significant differences in the indoleamine 2, 3-dioxygenase (IDO) expression were observed in those fibroblasts (Fig. 2A and Supplementary information). Moreover, the CAFs isolated from patients revealed a significantly increased secretion of Kyn (Fig. 2A and S1D) compared to normal fibroblasts HLFs, suggesting the potential role of Kyn metabolism in EGFR TKIs resistance in lung cancer cells. Current studies provided evidence that Kyn could mediate the activation of AhR signals, resulting in tumor development in several tumor types [17]. More importantly, the activation of AhR is also reported to be associated with drugs resistance development [18]. Herein, a further expression of AhR in A549 cells pre-cultured with CAFs and HFLs was examined. Notably, CAFs significantly up-regulated the AhR expression and promoted the nuclear localization of AhR in A549 cells. Kyn treatment in the HFLs co-culture system also promoted the nuclear entry of AhR (Fig. 2B), indicating that Kyn produced by CAFs could mediate the activation of AhR signals in lung cancer. To further explore the role of Kyn and AhR in lung cancer progression, Kyn and DMF (an AhR inhibitor) were added into the culture medium of A549 and Lewis. The CCK8 analysis proved that Kyn promoted the proliferation of A549 and Lewis, whereas blockade of AhR signals by DMF suppressed the pro-tumor effects induced by Kyn (Fig. 2C). Meanwhile, the cytotoxicity analysis implicated that Kyn treatment enhanced the drugs resistance of A549 (Fig. 2D) and Lewis (Fig. 2E) to Erl and Gef compared to PBS group. The similar results were found in DMF treated CAFs-tumor cells co-culture system (Fig. S1E and F). And no obvious influence of DMF was found on A549 or Lewis without Kyn treatment (Fig. S1G and H). Taken together, those results suggested that CAFs produced Kyn to facilitate lung cancer cells proliferation and EGFR TKIs resistance.

### Kyn mediates the AhR downstream AKT and ERK signals activation

**Fig. 3:**
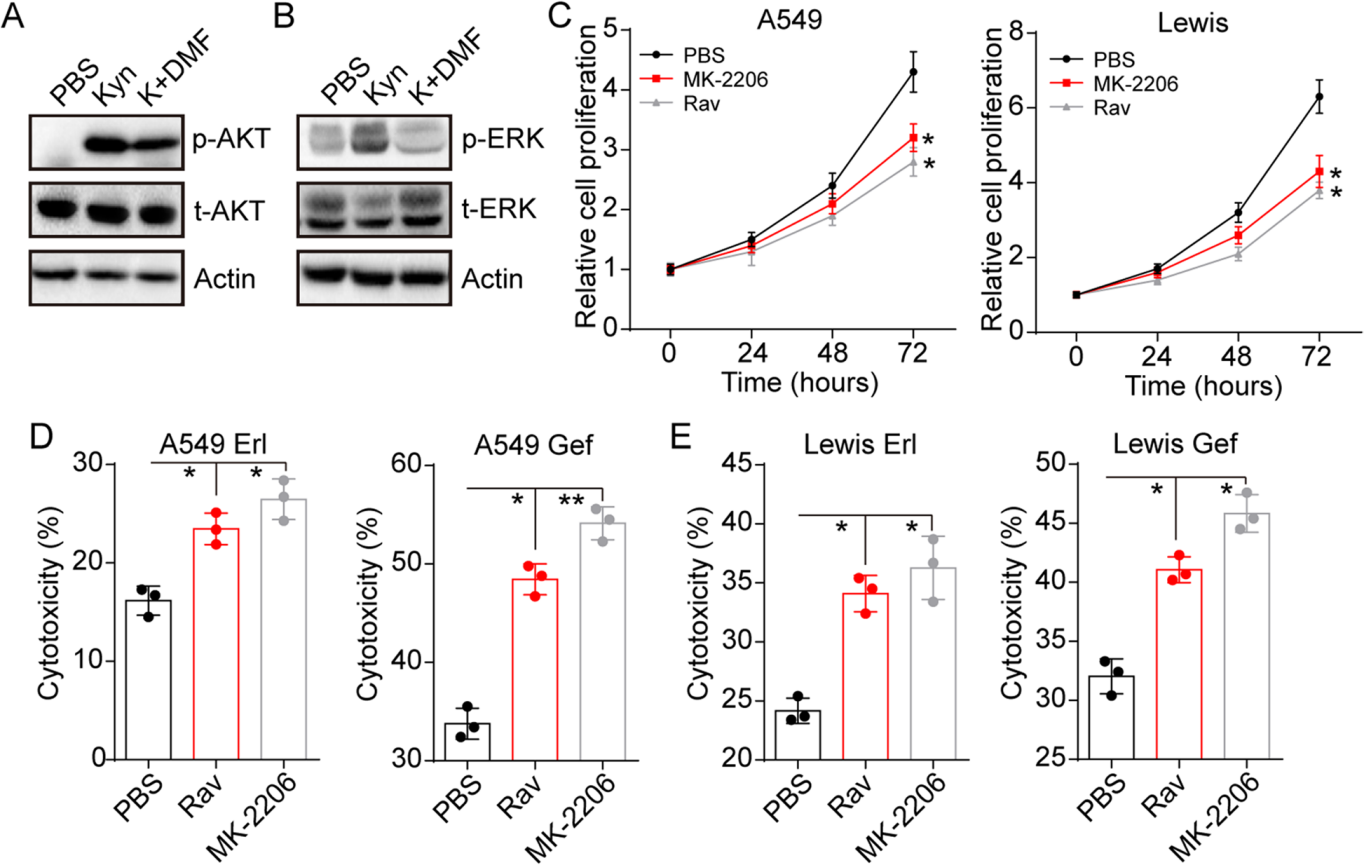
Kyn produced from CAFs mediates the activation of AKT and ERK signals in lung cancer cells. **A**, Western blotting of phosphorylated AKT, total AKT, and actin in A549 cells treated with PBS, Kyn, and Kyn combined with DMF (Kyn 100 μM, DMF 10 μM, 48 hours). **B**, Western blotting of phosphorylated ERK1/2, total ERK1/2, and actin in A549 cells treated with PBS, Kyn, and Kyn combined with DMF (Kyn 100 μM, DMF 10 μM, 48 hours). **C**, the relative cells proliferation of Kyn (100 μM) cultured A549 and Lewis treated with PBS, MK-2206 (10 nM), and Rav (5 nM). **D**, the cytotoxicity of Kyn (100 μM) cultured A549 treated with PBS, MK-2206 (10 nM), and Rav (5 nM) to Erl (5 μM, 48 hours) and Gef (10 μM, 48 hours). **E**, the cytotoxicity of Kyn (100 μM) cultured Lewis treated with PBS, MK-2206 (10 nM), and Rav (5 nM) to Erl (5 μM, 48 hours) and Gef (10 μM, 48 hours). * indicates *P* < 0.05. ** indicates *P* < 0.01. n.s. indicates no statistical significance

The aberrant activation of the nonreceptor tyrosine kinase Src-associated signaling pathways, such as PI3K/AKT and MEK/ERK signals, have been emerging as a determinant of multiple resistance occurrence in lung cancer cells. To examine the possibility of AhR-induced AKT or ERK signals activation, the expression of phosphorylated AKT and ERK1/2 was determined. Intriguingly, obvious up-regulation of phosphorylated AKT and ERK1/2 was found in Kyn treated A549 and Lewis’s cells compared to the control group, whereas blockade of AhR by DMF suppressed the up-regulation of AKT and ERK1/2 (Fig. 3A and B and S1I), suggesting that Kyn mediated the activation of AKT and ERK signals through AhR upregulation. To further determine the role of AKT and ERK signals in Kyn-associated tumor progression, AKT inhibitor MK-2206 and ERK1/2 inhibitor Rav to treat lung cancer cells cultured with a medium containing Kyn was used. Notably, the blockade of AKT or ERK signals efficiently suppressed the proliferation of A549 and Lewis cells in the presence of Kyn (Fig. 3C). Suppression of AKT or ERK signals also suppressed the resistance of lung cancer cells to EGFR TKIs (Fig. 3D and E). Those results suggested that Kyn up-regulated AKT and ERK signals to regulate EGFR TKIs resistance in lung cancer.

### Blockade of AhR signals reversed EGFR TKIs resistance in lung cancer

**Fig. 4:**
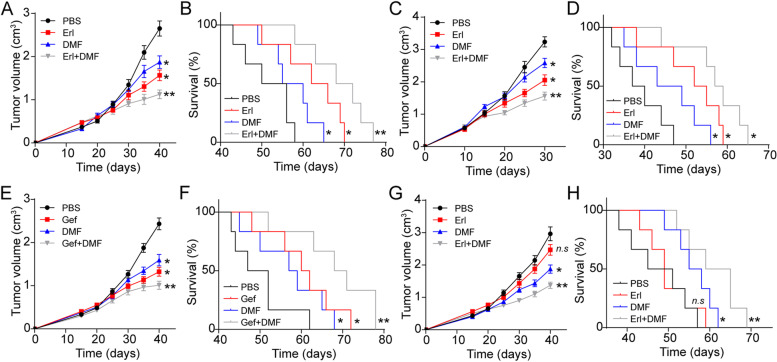
Blockade of AhR signals reverses EGFR TKIs resistance in lung cancer mice model. **A**, the tumor volume of A549 bearing mice treated with PBS, Erl, DMF, and Erl combined with DMF. **B**, the survival time of A549 bearing mice treated with PBS, Erl, DMF, and Erl combined with DMF. **C**, the tumor volume of Lewis bearing mice treated with PBS, Erl, DMF, and Erl combined with DMF. **D**, the survival time of Lewis bearing mice treated with PBS, Erl, DMF, and Erl combined with DMF. **E**, the tumor volume of A549 bearing mice treated with PBS, Gef, DMF, and Gef combined with DMF. **F**, the survival of A549 bearing mice treated with PBS, Gef, DMF, and Gef combined with DMF. **G**, the tumor volume of A549 (Kyn persistent treatment) bearing mice treated with PBS, Erl, DMF, and Erl combined with DMF. **H**, the survival time of A549 (Kyn persistent treatment) bearing mice treated with PBS, Erl, DMF, and Erl combined with DMF. * indicates *P* < 0.05. ** indicates *P *< 0.01. n.s. indicates no statistical significance

To examine the CAFs associated with EGFR TKIs resistance in vivo, a xenograft mouse model using A549 or Lewis cells was established. When the tumor reached 800 mm^3^, mice were treated with PBS, Erl and Erl combined with DMF. As a result, both Erl and DMF could suppress the tumor growth in vivo, whereas DMF treatment significantly improved the anticancer effects of Erl (Fig. 4A). Meanwhile, suppression of AhR signals efficiently prolonged the survival time of A459-bearing mice compared to the PBS group or Erl group (Fig. 4B), suggesting that blockade of AhR signals strengthened the anticancer effects of EGFR TKIs. The improved anticancer effects were also observed in Lewis bearing C57/BL mice (Fig. 4C and D). In line with the data, injection of DMF or Gef also succeeded to suppress the tumor growth and prolong the survival time of tumor-bearing mice compared to the PBS group, whereas combining treatment significantly extended the mice’s life (Fig. 4E and F). To establish the EGFR TKIs resistant tumor model, Kyn into A549-bearing mice was further intratumorally injected, following with Erl and DMF treatment. Intriguingly, Kyn significantly promoted the Erl resistance in A549 bearing mice, and DMF treatment revealed evident tumor-suppressive effects. More importantly, the addition of DMF reversed the drugs resistance and significantly improved Erl outcome in those tumor-bearing mice, indicating that DMF could reverse the drugs resistance induced by Kyn (Fig. 4G and H). Overall, these results implicated the therapeutic potential of AhR inhibitors in EGFR-associated lung cancer.

## Discussion

**Fig. 5:**
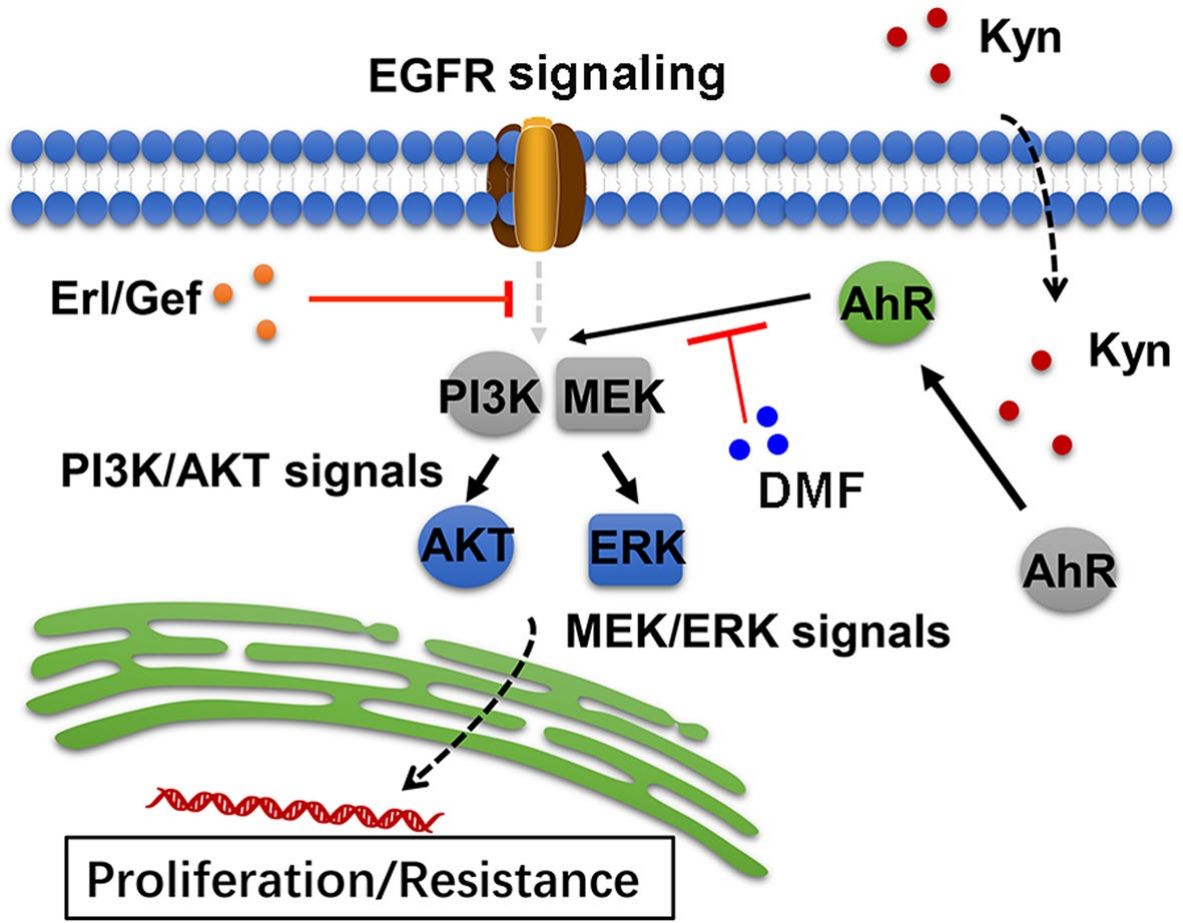
The schematic diagram of CAFs induced EGFR TKIs resistance in lung cancer

This study described a novel mechanism of EGFR TKIs resistance development in lung cancer. Here in this study, it is demonstrated that CAFs in tumor tissues could strengthen the lung cancer cell proliferation and EGFR TKIs resistance through the secretion of Kyn. Kyn produced by CAFs participated in the activation of AhR signals in tumor cells, thereby resulting in the downstream AKT/ERK signals activation (Fig. 5). These findings also provided evidence to suggest AhR signals coming into being in the tumor drugs resistance development process, which expands the role of AhR in the targeted therapeutic strategy for lung cancer treatment.

Increasing evidence suggested that tumor progression is bound up with the stromal and immune cells in the tumor microenvironment [19, 20]. Among those cells, CAFs are emerging as crucial participants in regulating tumor growth, cells migration, and tumor responses to clinical therapy [21]. Previous reports have implicated that CAFs could secret various cytokines or metabolites, which facilitated tumor growth or epithelial-mesenchymal transition, eventually resulting in cancer development [22, 23]. For instance, Subramaniam et al. suggested that CAFs could promote endometrial cancer growth through secretion of IL-6, leading to the STAT-3/c-Myc pathway activation in tumor cells [24]. More importantly, CAFs are prone to facilitate cells migration and cancer distant metastasis in breast cancer via secretion of CXCL12 [25]. Wei et al., also demonstrated that CAFs are capable of mediating gemcitabine resistance via the SDF-1/SATB-1 pathway in pancreatic cancer [26]. Those findings provided significant evidence to implicate the role of CAFs in tumor progression, and this study further described the role of CAFs in lung cancer EGFR TKIs resistance development, which is dependent on the secretion of Kyn in CAFs. Notably, Kyn involved immune suppression in the tumor microenvironment has been recognized as a critical determinant in immune-associated cancer therapy [27]. This study further determined that elevated expression of TDO2, instead of IDO1, mediated the Trp metabolism in CAFs, resulting in sustained Kyn production, which provides a novel sight in the role of IDO/TDO in stromal cells and tumor progression.

In addition to the response to the cytotoxicity of 2,3,7,8-tetrachlorodibenzo-p-dioxin (TCDD) and other extracellular pollutants, AhR also serves as an indispensable regulator in immune responses and tumor progression [28]. Merging evidence suggested that AhR is a key sensor allowing immune cells to adapt to environmental alternations, and the activity has been proved to be associated with autoimmune disorders and cancer [29]. Compelling studies have demonstrated that the expression of AhR is tightly correlated to the drug’s resistance development in several tumor types [30, 31]. A Dubrovska and his colleagues reported that CXCR4 activation could maintain the stem cell population in tamoxifen-resistant breast cancer cells through AhR signaling [32]. NC D’Amator provided evidence that the TDO2-AhR signaling axis might facilitate anoikis resistance and metastasis in triple-negative breast cancer [33]. Thereby, suppression of AhR signals by directly targeting AhR or upstream ITO/TDO signals is expected to mediate tumor regression. Indeed, the AhR inhibitor DMF exhibited efficient tumor-suppressive effects in this mice model and prolonged the overall survival of tumor-bearing mice. More importantly, testing AhR expression or Kyn level might help to predict the EGFR TKIs responses in lung cancer.

Given the limitations of previous reports, this study further described the role of CAFs in lung cancer EGFR TKIs resistance development. We expounded on the underlying mechanism of CAFs associated with EGFR TKIs resistance, which is dependent on a Kyn/AhR/AKT/ERK signaling pathway. Blockade of AhR efficiently improved the outcome of EGFR TKIs, which provides a novel strategy for clinical lung cancer treatment.

## Supplementary Information

**Additional file 1.**

## Data Availability

The datasets used and/or analyzed during the current study are available from the corresponding author on reasonable request.

## Abbreviations

CAFs: cancer associated fibroblasts
NSCLC: Non-small cell lung cancer
Kyn: kynurenine
TDO: tryptophan 2,3-dioxygenase
EGFR TKIs: epidermal growth factor receptor tyrosine kinase inhibitors
AhR: Aryl Hydrocarbon Receptor
Rav: ravoxertinib
Erl: erlotinib
Gef: gefitinib
IDO: indoleamine 2, 3-dioxygenase
TCDD: 2,3,7,8-tetrachlorodibenzo-p-dioxin
ELISA: Enzyme-linked immunosorbent assay
