# Supplementary material for: Cancer-associated fibroblasts strengthen cell proliferation and EGFR TKIs resistance through aryl hydrocarbon receptor dependent signals in non-small cell lung cancer
Source: BMC Cancer. 2022 Jul 13;22:764. doi: 10.1186/s12885-022-09877-7 (PMC9281029; doi:10.1186/s12885-022-09877-7)
Supplement: Supplementary file 1 — Additional file 1. [file 12885_2022_9877_MOESM1_ESM.docx]

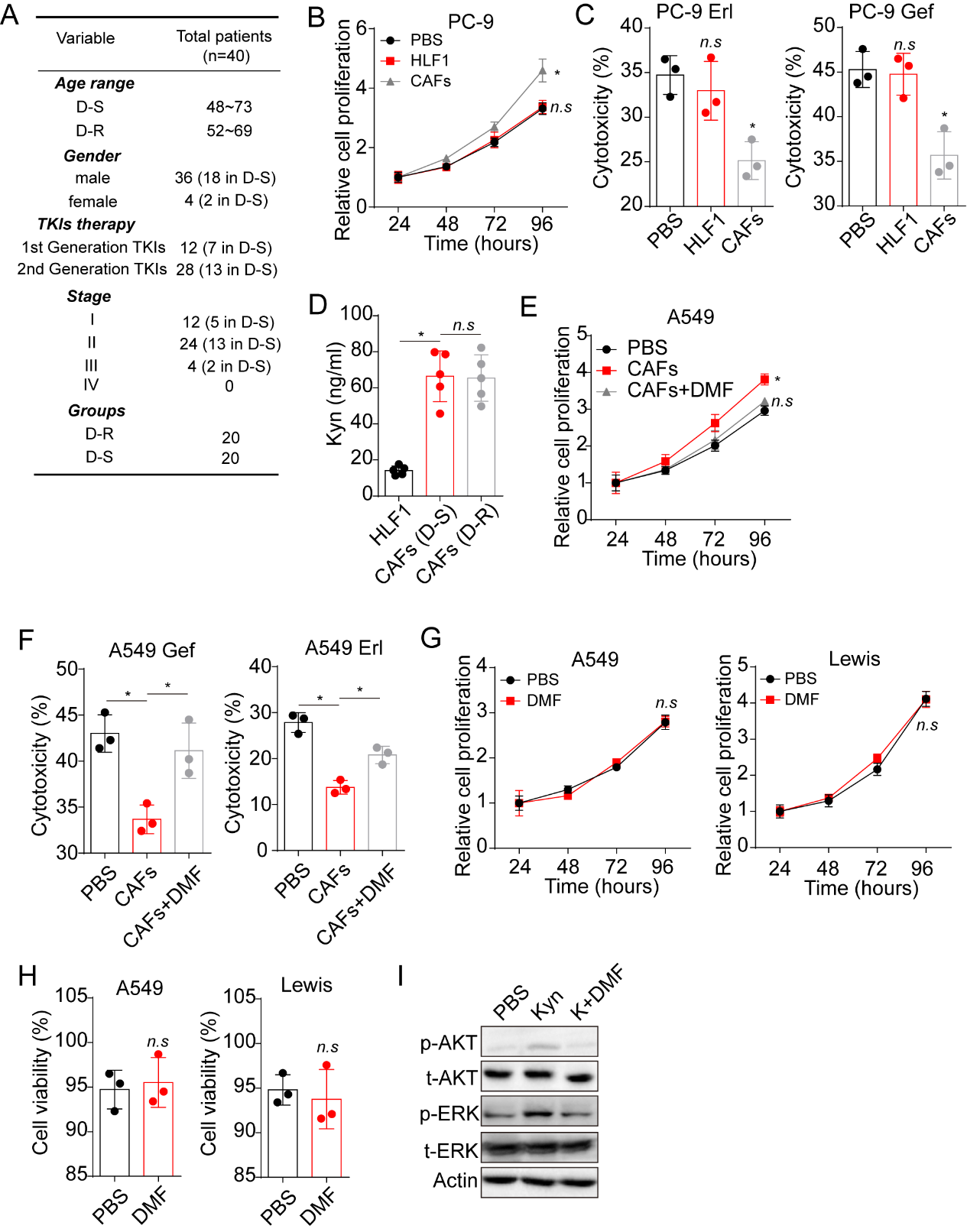


Supplementary figure 1

A, clinical characteristics of 40 NSCLC patients. B, the relative cells proliferation of PC-9 cells pre-cultured with PBS, HFL1, CAFs isolated from patients (fibroblast: tumor cells, 1:5, 48 hours). C, cytotoxicity of PC-9 cells pre-cultured with PBS, HFL1, CAFs to Erl (5 μM, 48 hours) and Gef (10 μM, 48 hours). D, Kyn quantification in supernatant of HFL1 or CAFs isolated from D-R/D-S patients (48 hours). E and F, A549 cells were co-cultured with CAFs (or not) and treated with DMF (10 μM, 48 hours, or not). The relative cell proliferation and cell apoptosis to Erl (5 μM, 48 hours) or Gef (10 μM, 48 hours) were determined. G, the relative cells proliferation of A549 and Lewis treated with PBS or DMF (10 μM). H, cytotoxicity of A549/Lewis cells pre-cultured with PBS or DMF (10 μM) to Erl (5 μM, 48 hours) and Gef (10 μM, 48 hours). I, the western blotting of phosphorylated AKT, total AKT, phosphorylated ERK1/2, total ERK1/2 and actin in Lewis cells treated with PBS, Kyn and Kyn combined with DMF (Kyn 100 μM, DMF 10 μM, 48 hours). * indicates P < 0.05. ** indicates P < 0.01. n.s. indicates no statistical significance.


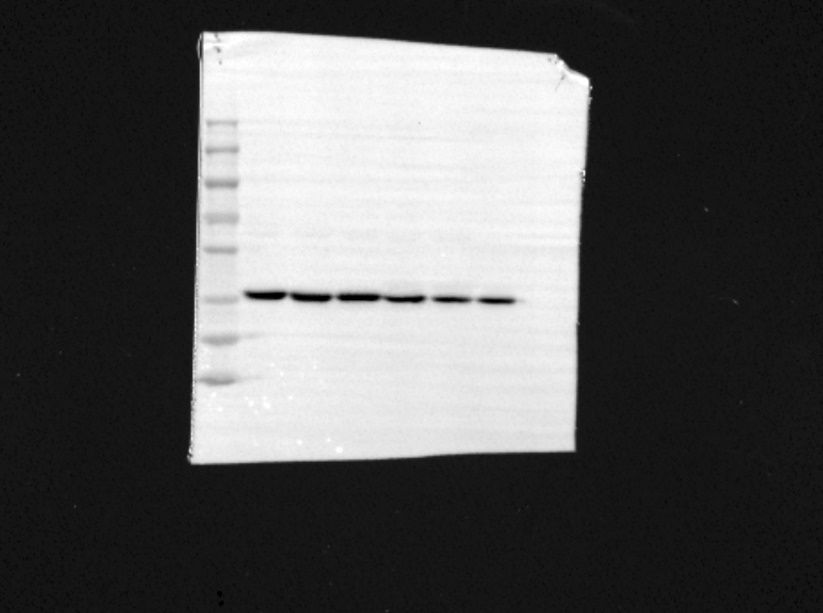


Actin (figure 3A right, figure 3B left)


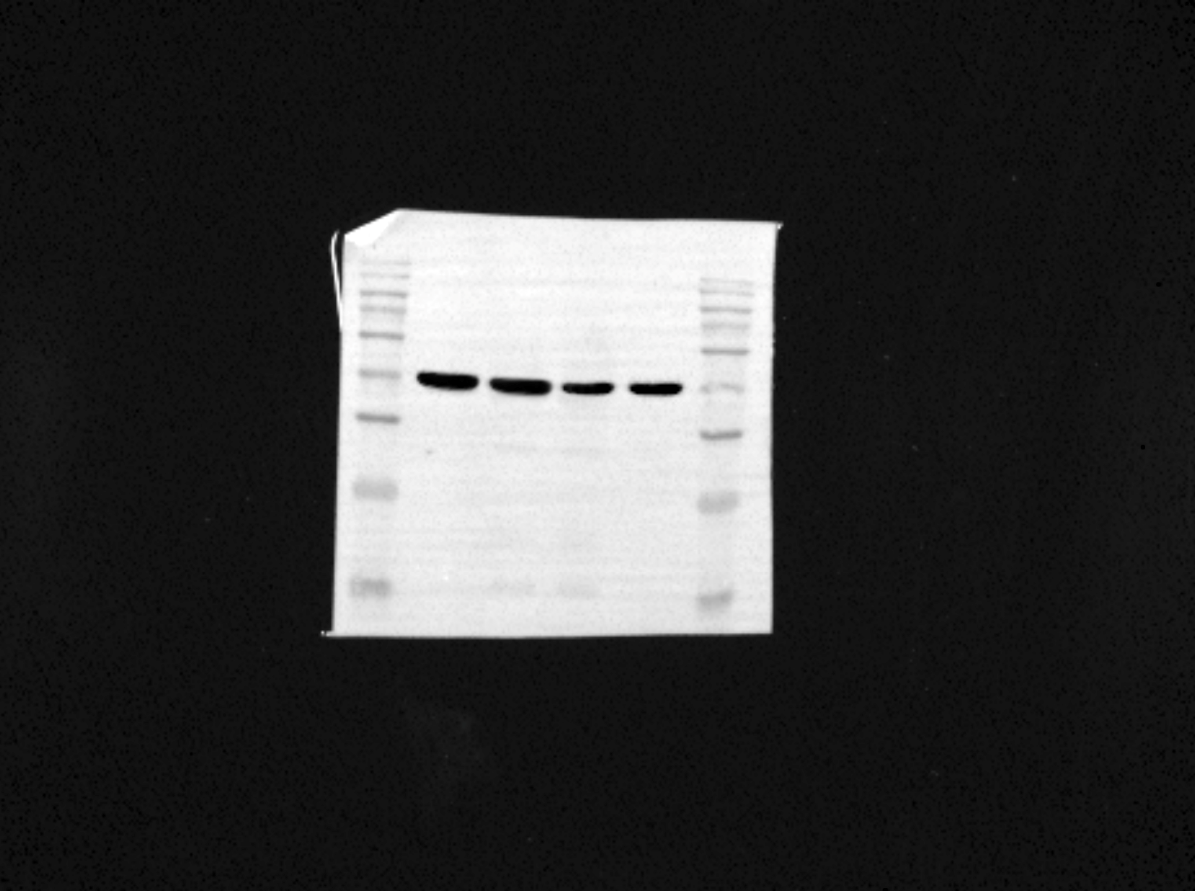


Actin (Fig. 1B, left) (Fig. 2A right)


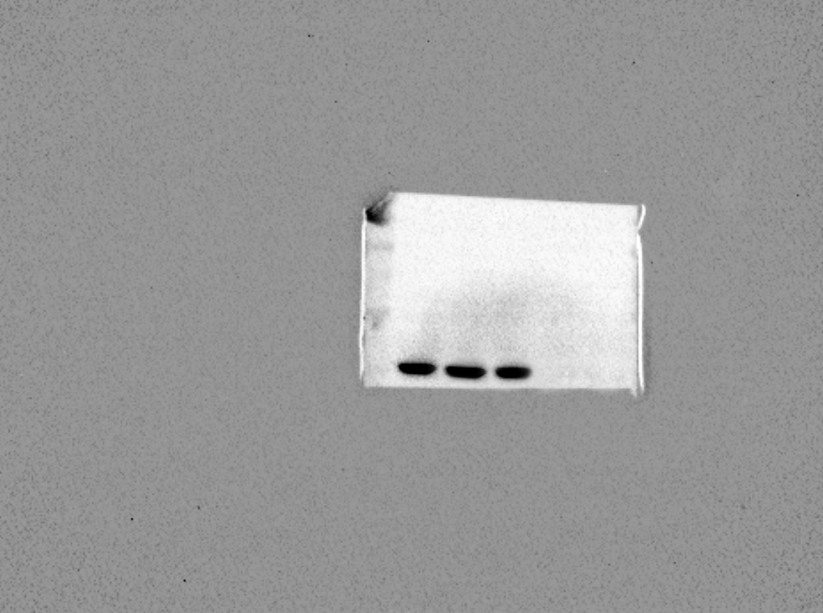


t-AKT (Fig. 3A)


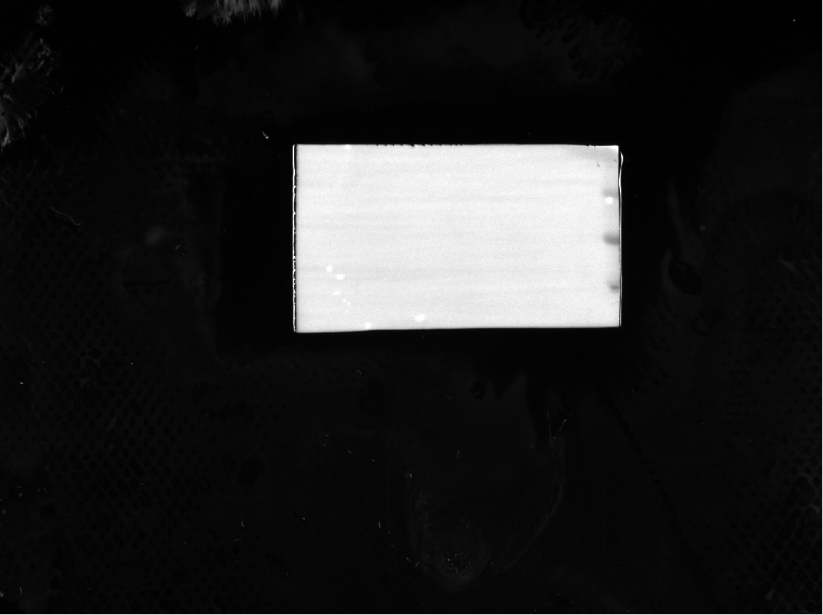


IDO1 (Fig. 2A)


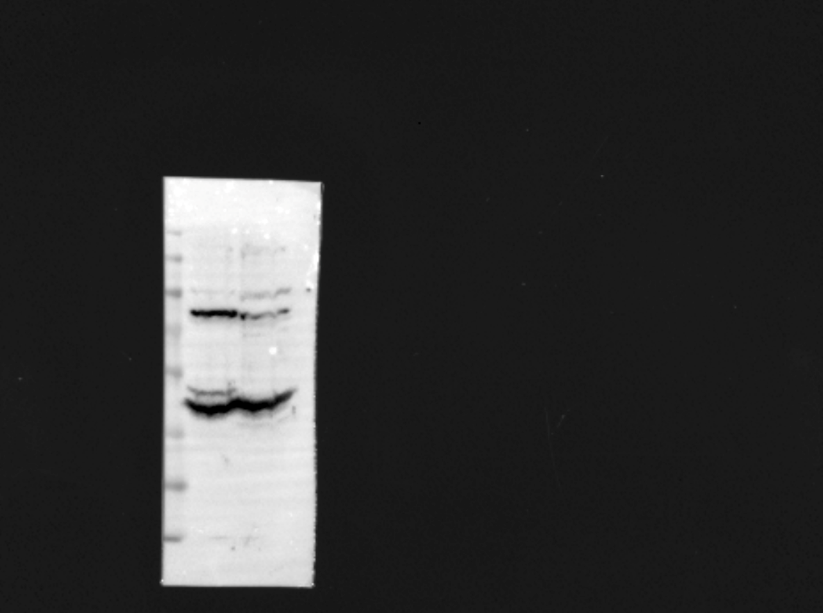


Kyn (Fig. 2A, mirror rotation)


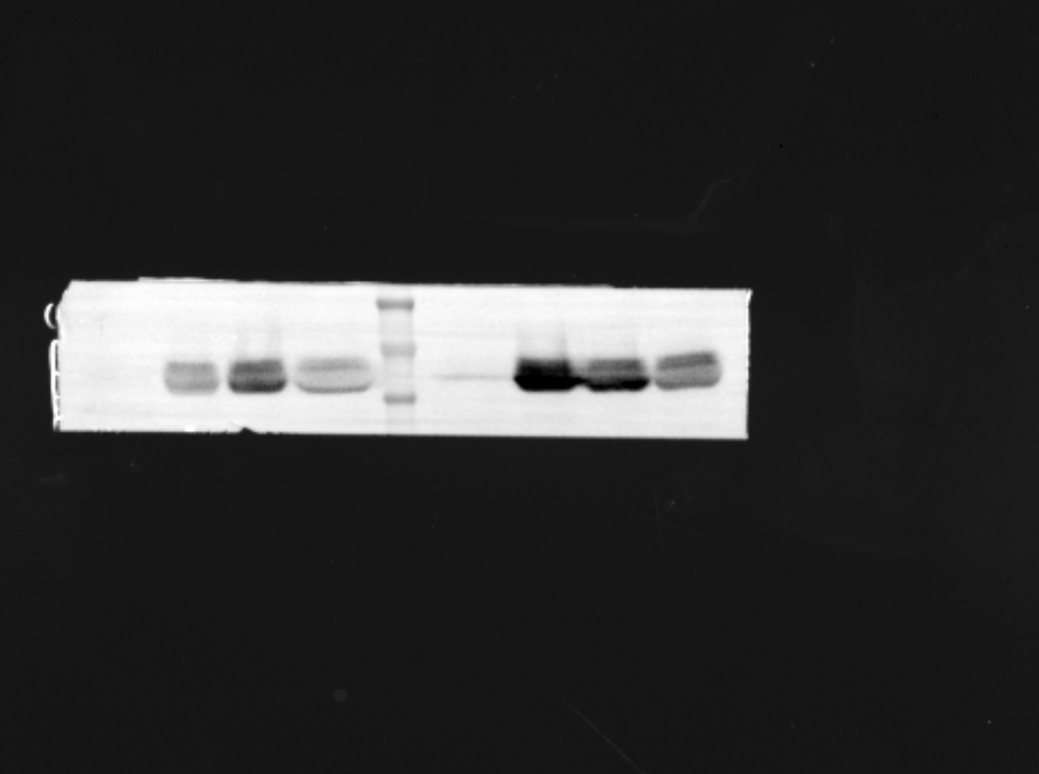


pERK Fig. 3B


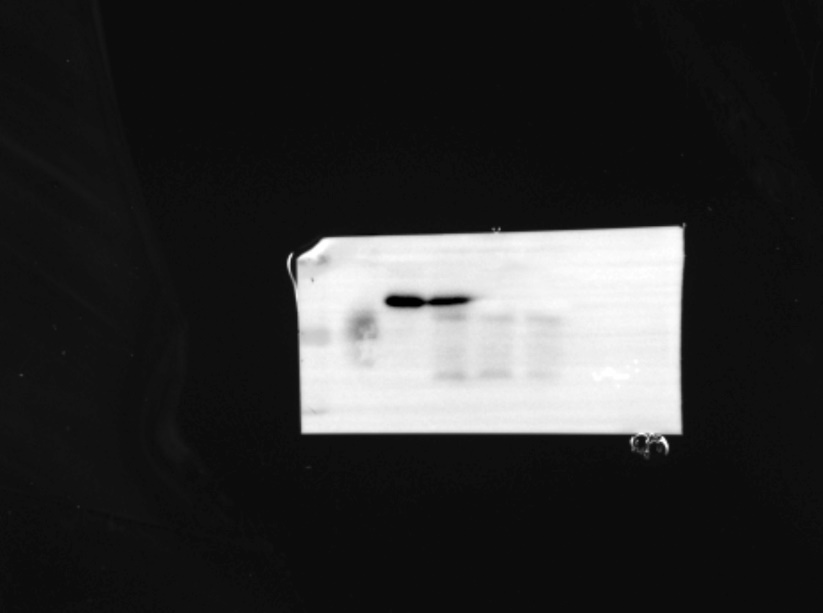


pAKT (Fig. 3A)


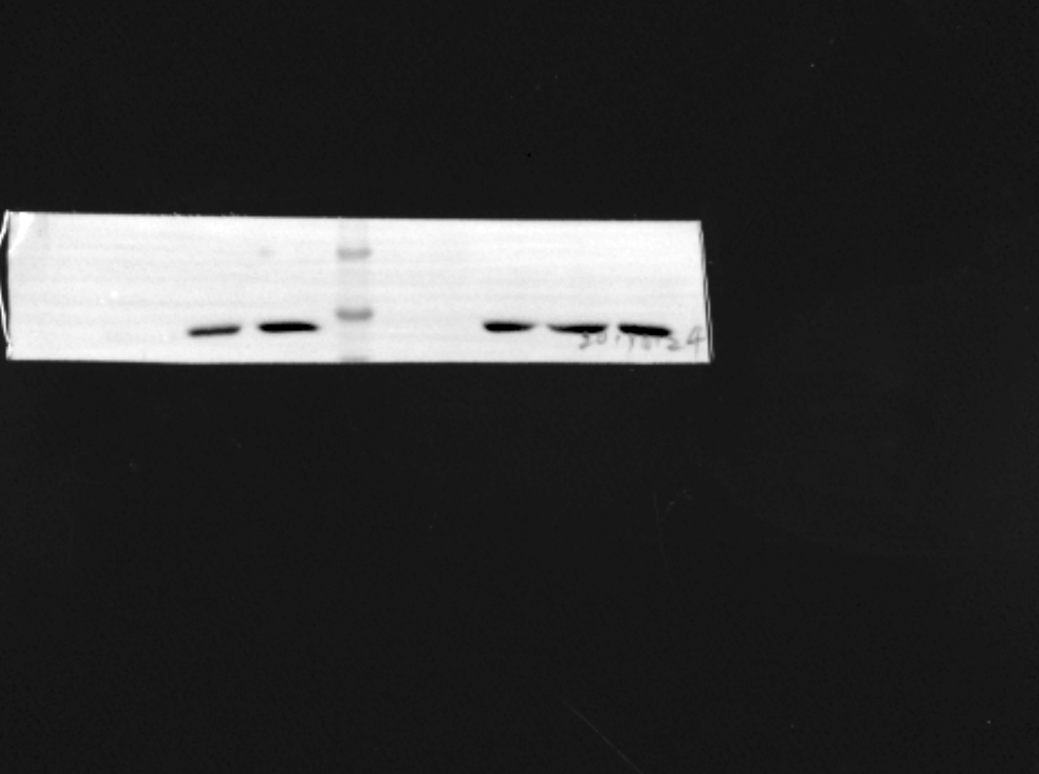


SMA Fig. 1B


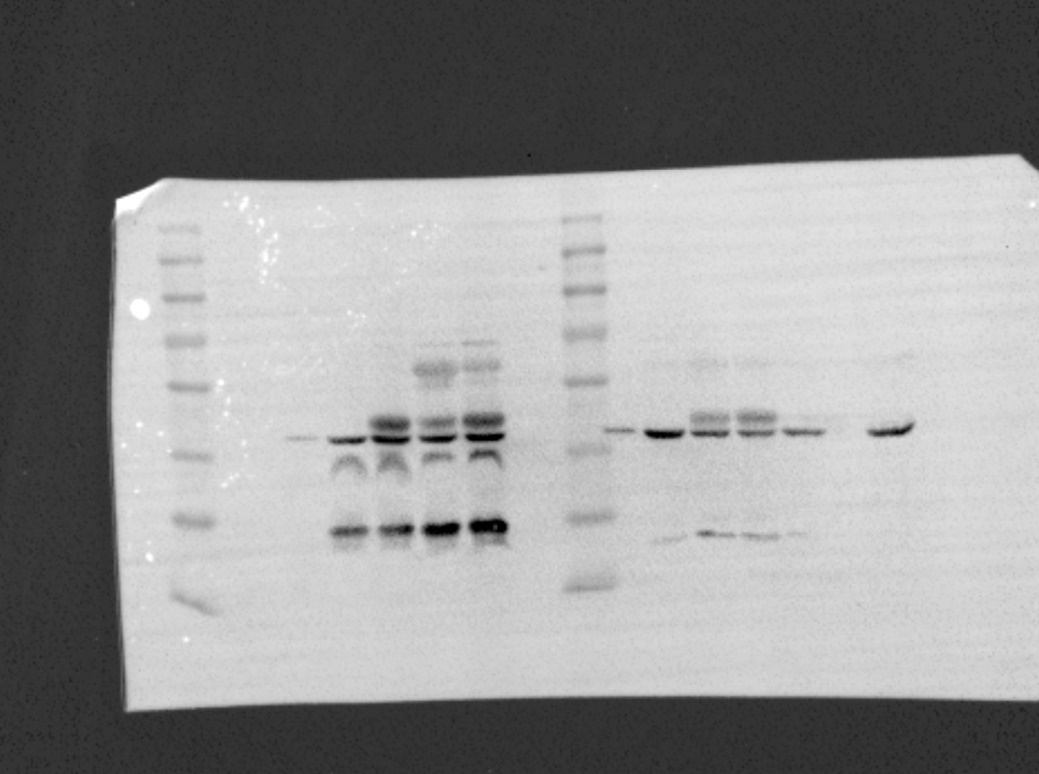


T ERK Fig. 3B


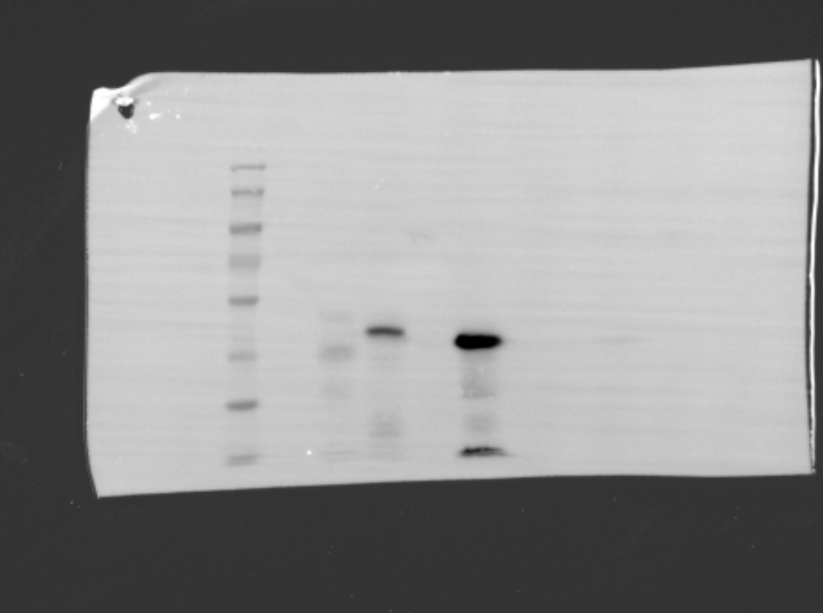


TDO2 Fig. 2A


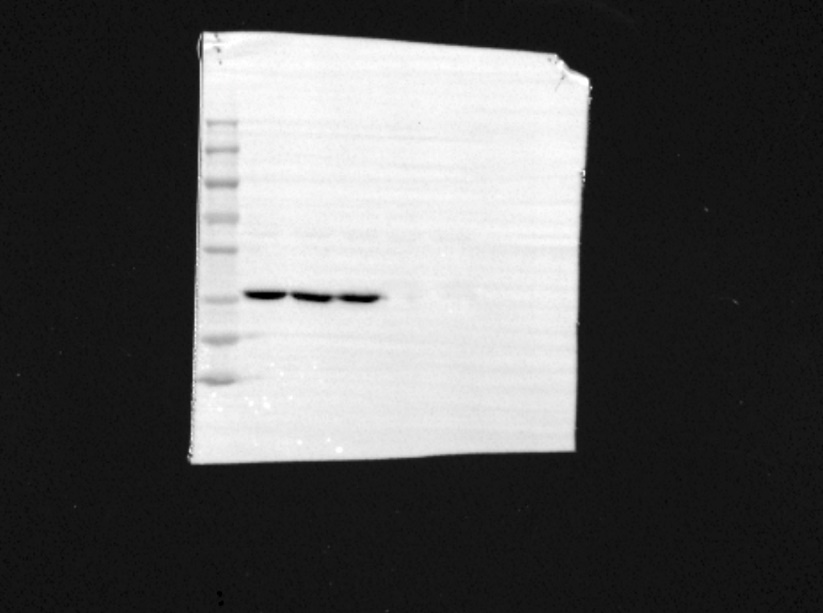


Fig. S1 ACTIN


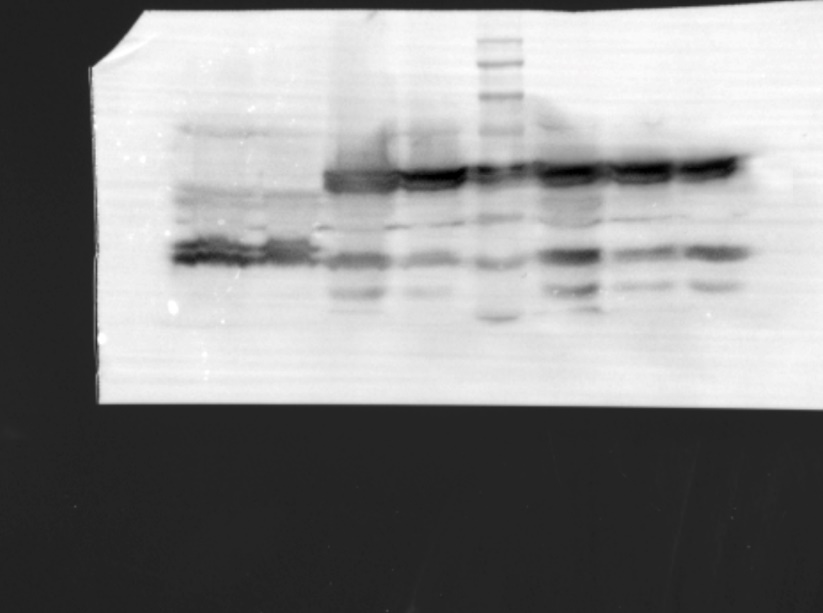


Fig. S1 t-ERK


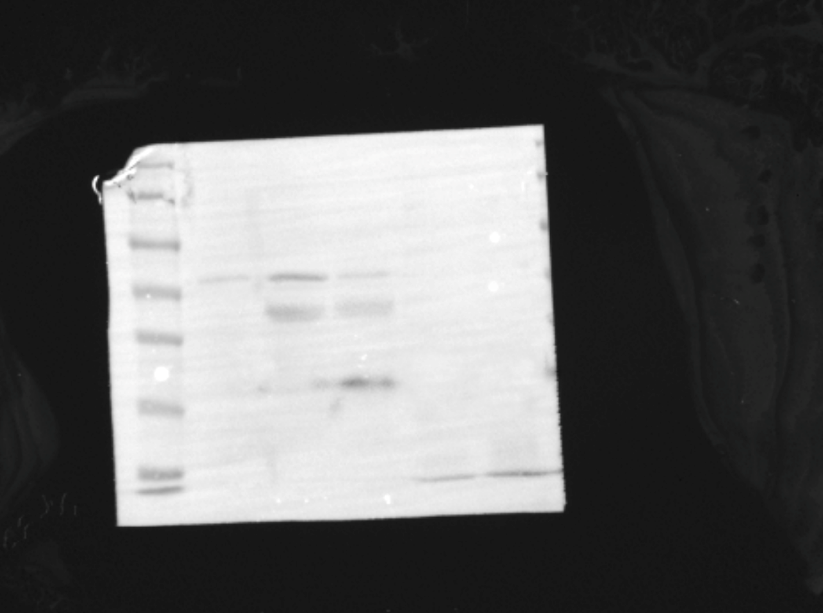


Fig. S1 p-AKT


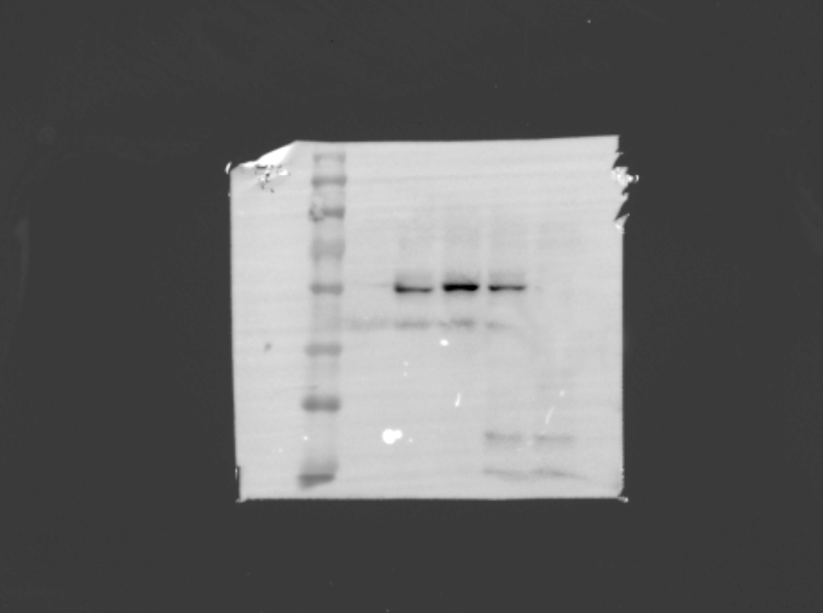


Fig. S1 p-ERK


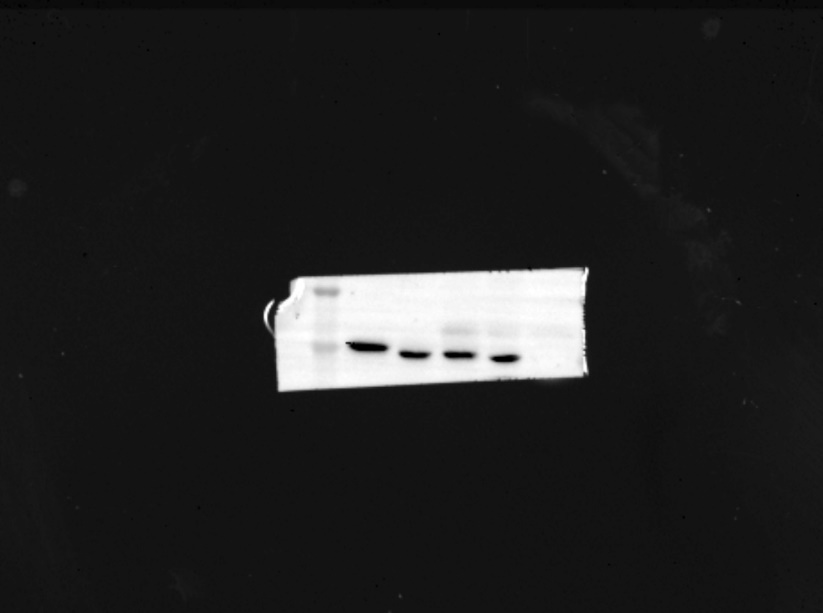


Fig. S1 t-AKT
